# Achieving flexible large-scale reactivity tuning by controlling the phase, thickness and support of two-dimensional ZnO†

**DOI:** 10.1039/d1sc04428a

**Published:** 2021-11-04

**Authors:** Le Lin, Zhenhua Zeng, Qiang Fu, Xinhe Bao

**Affiliations:** Shanghai Advanced Research Institute, Chinese Academy of Sciences Shanghai 201203 China; State Key Laboratory of Catalysis, Dalian Institute of Chemical Physics, Chinese Academy of Sciences Dalian 116023 China qfu@dicp.ac.cn; School of Physical Science and Technology, ShanghaiTech University Shanghai 201210 China; University of Chinese Academy of Sciences Beijing 100049 China; Davidson School of Chemical Engineering, Purdue University West Lafayette IN 47907 USA zeng46@purdue.edu

## Abstract

Tuning surface reactivity of catalysts is an effective strategy to enhance catalytic activity towards a chemical reaction. Traditional reactivity tuning usually relies on a change of the catalyst composition, especially when large-scale tuning is desired. Here, based on density functional theory calculations, we provide a strategy for flexible large-scale tuning of surface reactivity, *i.e.* from a few tenths of electronvolts (eV) to multiple eV, merely through manipulating the phase, thickness, and support of two-dimensional (2D) ZnO films. 2D ZnO films have three typical phases, *i.e.* graphene, wurtzite, and body-centered-tetragonal structures, whose intrinsic stability strongly depends on the thickness and/or the chemical nature of the support. We show that the adsorption energy of hydrogen differs by up to 3 eV on these three phases. For the same phase, varying the film thickness and/or support can lead to a few tenths of eV to 2 eV tuning of surface reactivity. We further demonstrate that flexible large-scale tuning of surface reactivity has a profound impact on the reaction kinetics, including breaking the Brønsted–Evans–Polanyi relationship.

## Abstract 2

Flexible large-scale reactivity tuning is achieved by manipulating the phase, thickness and support of two-dimensional ZnO, and a broken scaling relationship between adsorption and barrier is found *via* phase and termination engineering.

## Graphical abstract

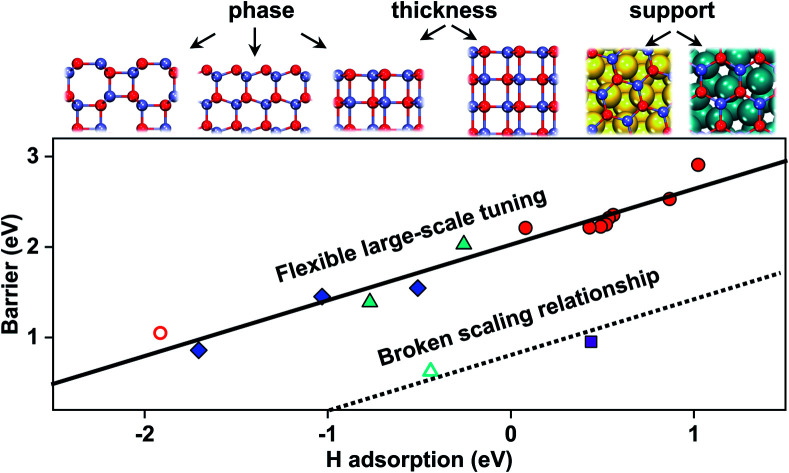

## Introduction

Surface reactivity of a catalyst, as characterized by adsorption energies of reaction intermediates, usually serves as an activity descriptor toward catalytic reactions through the scaling relationship between the energetics of reaction intermediates, and the Brønsted–Evans–Polanyi (BEP) relationship between the adsorption reactivity and reaction kinetics.^1–6^ Thus, tuning surface reactivity of catalysts presents a facile way to improve the activity of a catalytic reaction. As a consequence, a variety of strategies for reactivity tuning have been developed.

The most common approach for reactivity tuning is probably through changing the catalyst composition. This approach may lead to multiple eV changes in adsorption energies of reaction intermediates.^7,8^ Inevitably, the large-scale tuning, induced by the distinct chemical nature of a variety of compounds, also leads to large error bars in the scaling relationship and the BEP relationship, and consequently a low accuracy in the prediction of new catalysts with improved performance.^6,9^ Thus, to reduce these error bars, reactivity tuning using compounds with similar chemical nature or ideally using the same compound is desired.

The first strategy in this category is through varying the local coordination environment of a compound. This approach can lead to up to an eV level change in the adsorption energy of reaction intermediates, and smaller error bars in the scaling relationship, especially when the generalized coordination number has been considered.^10–13^ However, it is highly infeasible to synthesize catalysts with a uniform coordination environment, rather a mixture of sites with a large range of coordination numbers, which makes the actual reactivity tuning a challenge. The second strategy is through manipulating the crystal phase of a compound.^14–16^ While a change in the crystal phase may have a smaller effect on reactivity tuning, *e.g.* <0.5 eV, than that of the local coordination environment, it still has a significant impact on the catalytic activity due to structure sensitivity, as demonstrated by Liu *et al.* regarding hexagonal close-packed (hcp) Co *versus* face centered-cubic (fcc) Co for Fischer–Tropsch (FT) synthesis,^17–19^ and Wang *et al.* regarding cubic In_2_O_3_*versus* hexagonal In_2_O_3_ for the reverse water gas shift (RWGS) reaction.^20^ However, the desired crystal phases are usually metastable, which may lead to phase transition issues during catalytic reactions. The third strategy is through controlling the bond length of the active sites and associated strain.^21–24^ This method could lead to the fine tuning of surface reactivity (*e.g.* <0.1 eV) and the optimization of catalytic activity toward optimal values.^25^ However, engineering precise strain remains a great challenge. Also, there is no existing strategy that could bridge all the above scales using a single compound catalyst, especially large-scale tuning achieved through changing the catalyst composition. Such flexible and large-scale tuning is crucial for the design of multi-functional catalysts for a variety of applications.

Here, by employing density functional theory (DFT) calculations and using two-dimensional (2D) ZnO as a typical example, we demonstrate the possibility of flexible and large-scale tuning of surface reactivity by manipulating the phase, thickness, and support of ZnO films. In detail, using H adsorption energy as a descriptor of surface reactivity, we show that the tuning of surface reactivity is up to 3 eV between the nonpolar graphene type phase and polar wurtzite type phase of free-standing ZnO. The tuning of surface reactivity also leads to a corresponding change in reaction kinetics. Using Au(111) and Ru(0001) substrates as examples, we further demonstrate that small-scale to intermediate-scale reactivity tuning can be achieved by forming inverse ZnO/substrate structures and by varying the ZnO thickness. Further, we find that varying the chemical nature of the support also leads to a change of the scaling relationship between surface reactivity and reaction kinetics, *i.e.* breaking the BEP relationship.

## Results and discussion

**Fig. 1:**
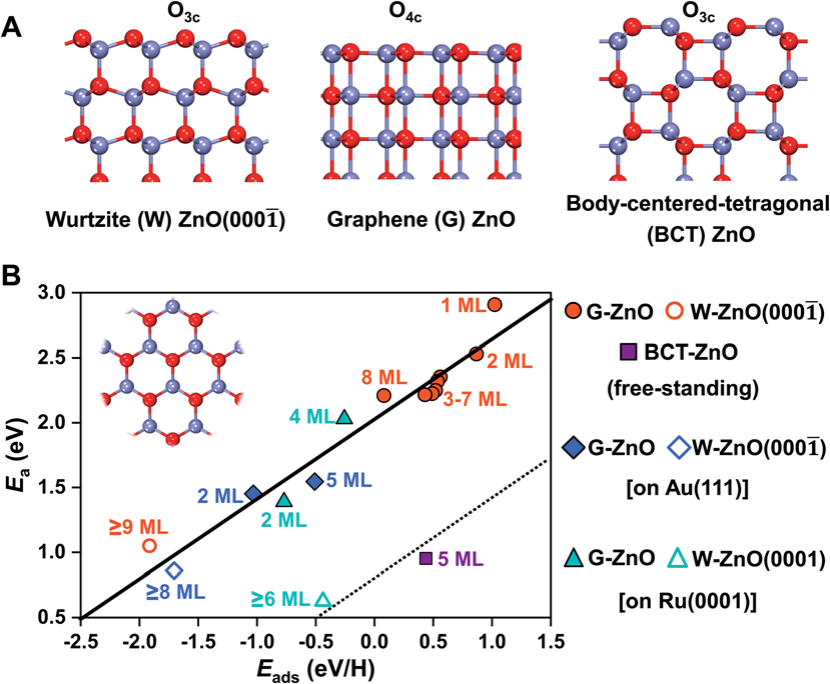
Surface reactivity of 2D ZnO films. (A) Side view of three typical free-standing 2D ZnO films, including the wurtzite type (W-), graphene type (G-), and body-centered-tetragonal type (BCT-) phases. Three and four-coordinated O atoms on the surface are marked as O_3c_ and O_4c_, respectively. (B) Scaling relationship (*y* = 0.62*x* + 2.03, *R*^2^ = 0.95) between the hydrogen adsorption energy and activation barrier on the surfaces of free-standing ZnO, ZnO/Au(111), and ZnO/Ru(0001), including the G-ZnO, W-ZnO, and BCT-ZnO phases. The inset shows the top view, which is the same for all three phases. Zn: light blue; O: red.

2D ZnO films commonly have three different phases, including the wurtzite type (W-ZnO), graphene type (G-ZnO), and body-centered-tetragonal type (BCT-ZnO) structures (see Fig. 1A). While they all have hexagonal symmetry within the plane (see the inset in Fig. 1B), there are notable differences in the detailed atomic structures, including the coordination of surface O atoms *e.g.* three-coordinated out-of plane O_3c_ in W-ZnO and four-coordinated in-plane O_4c_ in G-ZnO. The 2D W-ZnO phase is essentially with a polar wurtzite ZnO{0001} surface, including Zn-terminated W-ZnO(0001) and O-terminated W-ZnO(0001̄). The 2D G-ZnO phase is evolved from the W-ZnO phase but with coplanar Zn and O atoms and thus a nonpolar phase. The 2D BCT phase can be obtained by truncating the (100) facet of the BCT bulk, which is also nonpolar but more corrugated than that of the G-ZnO phase.

As shown in Fig. 1B, these distinct geometric features, along with the consideration of the film thickness and supports, lead to a flexible large-scale change in surface reactivity that was usually achieved only through multiple compounds with distinct chemical nature. More specifically, the resulting difference in surface reactivity is from a few tenths of eV up to 3 eV, as indicated by hydrogen adsorption. Described by the BEP relationship, these differences in surface reactivity also have a profound impact on the reaction kinetics. Below, we will perform a detailed analysis regarding the influence of the phase, thickness, and support on the surface reactivity of 2D ZnO films.

### Surface reactivity of free-standing ZnO films

**Fig. 2:**
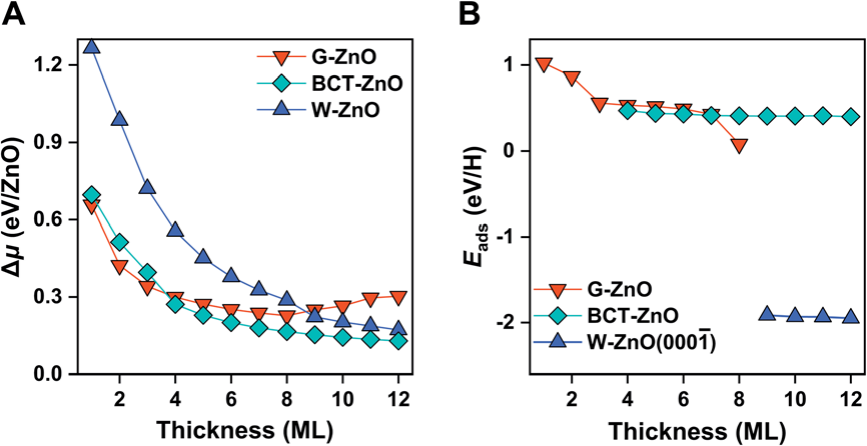
Stability and surface reactivity of free-standing ZnO films. (A) The relative chemical potential (Δ*μ*) of free-standing ZnO films with respect to wurtzite bulk ZnO, including G-ZnO, W-ZnO, and BCT-ZnO phases. (B) H adsorption energy on the surface O sites for the G-ZnO, W-ZnO(0001̄), and BCT-ZnO films.

Before moving on to the surface reactivity of free-standing 2D ZnO, we briefly discuss the stability of each phase and plausibility of phase engineering. Fig. 2A shows the relative chemical potential (Δ*μ*) of 2D ZnO films with respect to the bulk ZnO wurtzite-structure. For wurtzite ZnO[0001] truncation, films with thicknesses of <9 ML are evolved into the G-ZnO phase, while those of ≥9 ML prefer the W-ZnO phase, showing thickness-dependence of phase stability.^22,26,27^ For BCT ZnO[100] truncated films, they are less stable than G-ZnO when the thickness is <4 ML, but more stable than both G-ZnO and W-ZnO at a thickness of ≥4 ML.^26^ Thus, tuning the crystal face index and film thickness represents two strategies to engineer 2D ZnO films with different phases. It is worth noting that all three phases have been observed experimentally.^28–31^ As different phases usually have distinct reactivity, engineering these phases may lead to notable tuning of surface reactivity of ZnO. To verify this hypothesis, below we study hydrogen adsorption on these phases.

For G-ZnO, the adsorption energy (*E*_ads_) of atomic H on the O sites is in the range of 0.08 to 1.02 eV with the film thickness ranging from 1 to 8 ML (Fig. 2B and S1†), but for W-ZnO films with thicknesses of 9–12 ML, *E*_ads_ is around −1.9 eV and nearly independent of the film thickness, which indicates a 2–3 eV strengthened adsorption in comparison with that on G-ZnO. Such a big difference in hydrogen adsorption indicates that engineering these phases through varying the film thickness indeed can lead to significant tuning of surface reactivity of ZnO films. For BCT-ZnO films, *E*_ads_ is also nearly independent of the film thickness and is 0.44 ± 0.03 eV for 4–12 ML. Considering that the hydrogen adsorption energy is in the range of 0.56 to 1.02 eV for G-ZnO with the thickness ranging from 1 to 3 ML, the hydrogen adsorption energy is stabilized by 0.12–0.58 eV after phase transition from G-ZnO to BCT-ZnO. These results indicate that engineering G-ZnO and BCT-ZnO phases through varying the film thickness may lead to modest tuning of the surface reactivity of ZnO.

Based on the BEP relationship, tuning of the reaction energy would lead to a corresponding change in the reaction barrier.^7,32^ We thus infer that tuning of the H adsorption energy may lead to a corresponding change in the kinetic barrier of H-involved reactions, such as H_2_ dissociation and C–H scission. To verify the assumption, we have calculated H_2_ dissociation barriers on G-ZnO and W-ZnO. Indeed, the barrier (*E*_a_) of H_2_ dissociation on the surface O sites of W-ZnO(0001̄) is 1.04 eV, which is 1.3–1.4 eV lower than that on G-ZnO (Fig. 1, S2 and S3†). We analyse the strain evolution (*vs.* the wurtzite bulk) of 1–10 ML films, as shown in Fig. S2C.† For 1–8 ML G-ZnO, strain shows an increasing trend as the thickness increases, indicating that *E*_ads_ (the red line in Fig. 2B) shows a scaling relationship with the strain (Fig. S2D†). Such tensile strain elongates the Zn–O bond and renders enhancement of hydrogen adsorption and activation. On the other hand, for 9–10 ML W-ZnO with the surface geometry changing, the exposed surface O_3c_ sites are more active than O_4c_ in G-ZnO, which is favourable for activation of H_2_ beyond the strain effect.

In heterogeneous catalysis, 2D ZnO films are usually supported on the metal substrate by forming inverse oxide/metal catalysts.^33^ Using G-ZnO and W-ZnO as examples, the influence of the metal substrate on the surface reactivity of ZnO films is studied. We choose Au(111) and Ru(0001) surfaces as two representative supports with distinct chemical nature. For the BCT-ZnO phase, owing to its non-commensurate issue with these hexagonal substrates, it is no longer discussed below.

### Surface reactivity of Au(111)-supported ZnO films

**Fig. 3:**
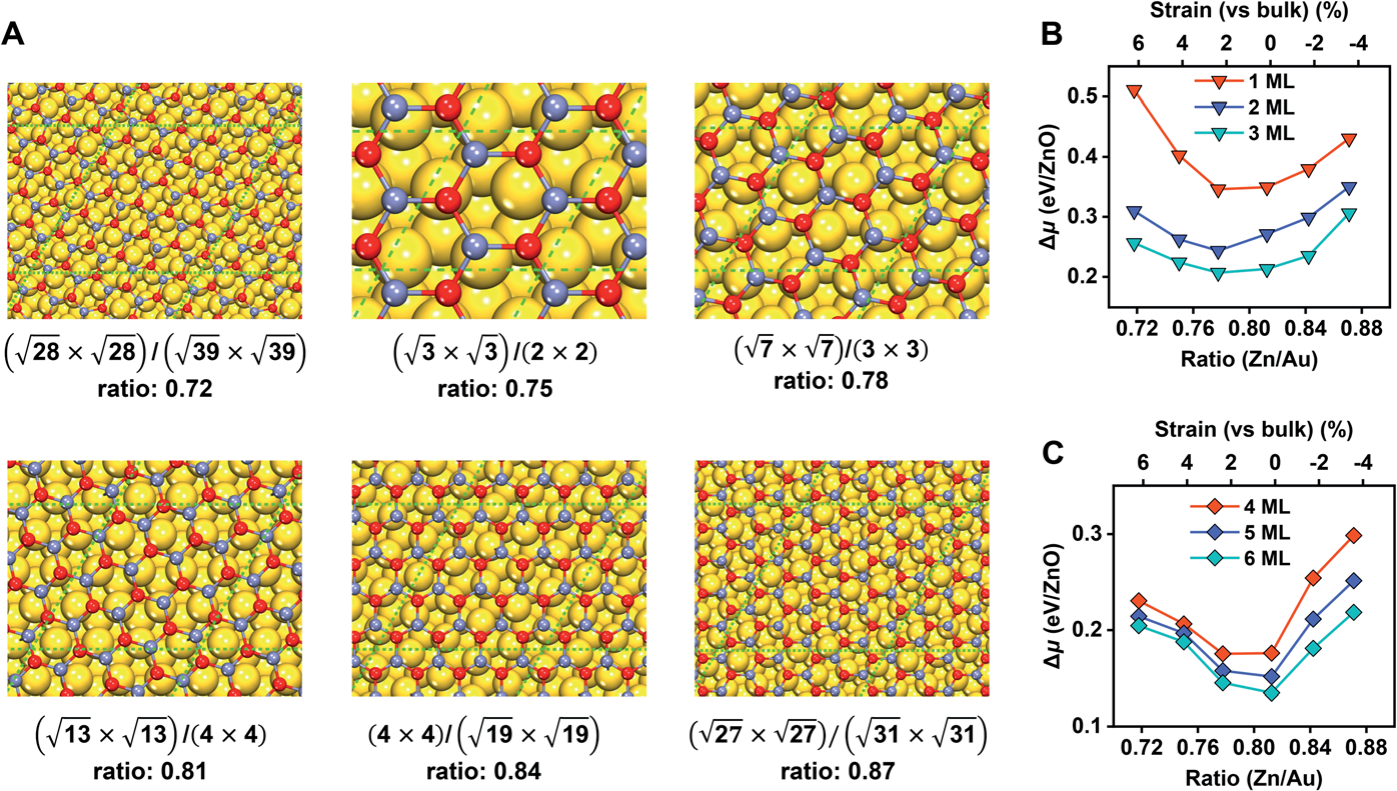
Interface models and the energetics of G-ZnO films on Au(111). (A) Moiré patterns of monolayer ZnO films supported on Au(111). Supercells of the film and substrate, and the interface Zn/Au ratios are given for each structure. The green dashed lines denote the boundaries of the unit cells. (B and C) The relative chemical potential (Δ*μ*), with respect to the bulk wurtzite ZnO and the Au(111) substrate, as a function of the interface Zn/Au ratio (the lower *x*-axis) and the intrinsic strains on the ZnO overlayer (the upper *x*-axis), for 1–3 ML (B) and 4–6 ML (C) ZnO films. O: red; Zn: light blue; Au: golden.

Before moving on to the surface reactivity of ZnO/Au(111), we first determine the stability of each phase (G-ZnO or W-ZnO) with respect to the strain, film thickness, and surface termination (*i.e.* O- or Zn-termination for W-ZnO). For G-ZnO, we consider a series of moiré patterns with the interface Zn/Au ratios ranging from 0.72 to 0.87 with an interval of ∼0.03 and with a film thickness of 1–6 ML (Fig. 3 and Table S1†). These ratios correspond to 6.4% to −3.4% of strain on ZnO overlayers (*vs.* the wurtzite bulk) with an interval of ∼2%.^34^ By comparing the relative chemical potential (Fig. 3B and C), we obtained the following trends. For the G-ZnO films with thicknesses of 1–3 ML, the most favourable interface is the (√7 × √7)/(3 × 3) structure with an interface Zn/Au ratio of 0.78 and a strain of 2.2%. It is worth noting that such an interface Zn/Au ratio is very close to the experimental value of 0.77 for ultrathin ZnO on Au(111).^35^ For the films with thicknesses of 4–6 ML, the (√13 × √13)/(4 × 4) structure with an interface Zn/Au ratio of 0.81 and near zero strain becomes more favourable, suggesting a trend toward the bulk lattice.

For each most favourable interface of G-ZnO/Au(111), Δ*μ* decreases with the increasing film thickness, *i.e.* from 0.35 eV for 1 ML ZnO to 0.13 eV for 6 ML ZnO, which is similar to that of free-standing G-ZnO. On the other hand, the difference between the (√7 × √7)/(3 × 3) and (√13 × √13)/(4 × 4) structures in Δ*μ* is much less sensitive to the film thickness (within 0.05 eV). Below, we used the (√13 × √13)/(4 × 4) structure to study the stability of G-ZnO with a thickness of >6 ML and the stability of W-ZnO.

**Fig. 4:**
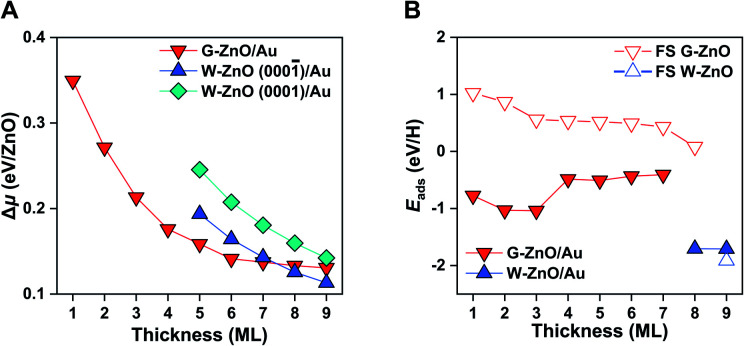
Stability and surface reactivity of ZnO/Au(111). (A) The relative chemical potential (Δ*μ*) of the ZnO films with different thicknesses on Au(111). (B) Hydrogen adsorption energy on ZnO/Au(111) *versus* the film thickness. As a comparison, those on free-standing ZnO films are also given in open triangles.

For both G-ZnO and W-ZnO on Au(111), Δ*μ* decreases with the increasing film thickness (Fig. 4A and S4†), denoting an increased stability. For W-ZnO, the absolute stability also depends on the surface termination, *i.e.* the (0001̄) surfaces with O termination are about 0.04 eV/ZnO more stable than the (0001) surfaces with Zn termination. Similar to free-standing films, the G-ZnO phase is more stable for thin films, while the W-ZnO phase is more stable for thick films, with the critical point between 7 and 8 ML, which is 1 ML thinner than that for free-standing films. Therefore, for ZnO/Au(111) it is also possible to tune surface reactivity through engineering phases which is controlled by varying the film thickness.

Indeed, G-ZnO/Au and W-ZnO/Au have a large difference in reactivity, as characterized by the H adsorption energy, though the magnitude is smaller than that of free-standing films (Fig. 4B). More specifically, the stabilization of hydrogen adsorption on W-ZnO/Au(111) is in the range of 0.7–1.3 eV in comparison with that on G-ZnO/Au(111), while the corresponding stabilization is 2–3 eV on the free-standing films. There are two reasons for the reduced difference between two phases. First, on G-ZnO/Au(111) films *E*_ads_ is in the range of −0.41 to −1.04 eV, which is 1–2 eV lower than that on free-standing G-ZnO. Such a strong stabilization can be ascribed to more corrugated surface atoms of G-ZnO/Au(111) than those of free-standing G-ZnO. Second, there is ∼0.2 eV weakening of hydrogen adsorption on W-ZnO/Au(111), in comparison with that on free-standing W-ZnO.

These changes in the hydrogen adsorption energy have two important implications for the surface reactivity and reactivity tuning. In comparison to free-standing ZnO, ZnO/Au(111) results in more moderate binding of hydrogen on the surface. In addition to large-scale reactivity tuning (*e.g.* >1 eV), a small (*e.g.* 0.1 eV) to intermediate-scale (*e.g.* 0.5 eV) tuning can be achieved by varying the film thickness of G-ZnO/Au(111). Therefore, the presence of the Au substrate leads to a broad range of reactivity tuning of each individual phase, in addition to the finer reactivity tuning through phase transformation. As dictated by the BEP relationship, the tuning of the surface reactivity suggests corresponding changes in the kinetic barrier of H-involved reactions, as demonstrated by H_2_ dissociation. More specifically, the presence of the Au substrate leads to a barrier of H_2_ dissociation varying by ∼0.7 eV.

Thus, the presence of the metal substrate has a substantial influence on the surface reactivity and associated reaction kinetics. As a noble metal, Au interacts weakly with the ZnO films,^22,32,36^ in comparison with other transition metals, *e.g.* Ru. Below, we use Ru(0001) as an active surface to gain insights into the influence of the chemical nature of the support on the surface reactivity and the reactivity tuning of ZnO films.

### Surface reactivity of Ru(0001)-supported ZnO films

**Fig. 5:**
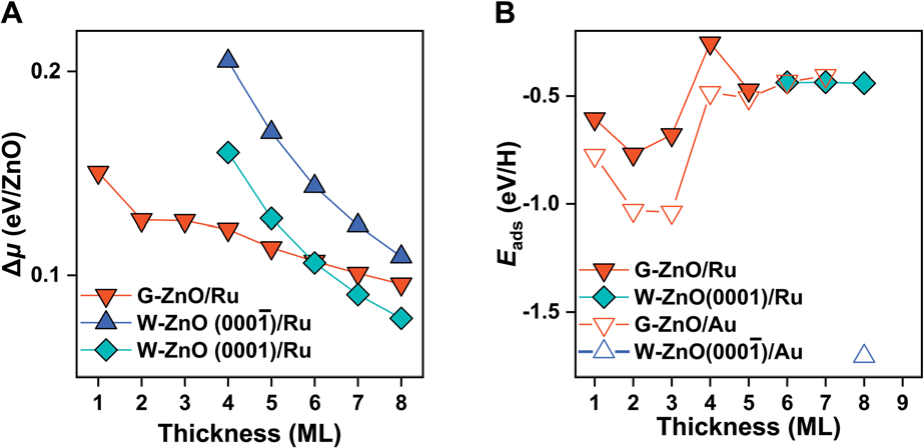
Stability and surface reactivity of ZnO/Ru(0001). (A) The relative chemical potential (Δ*μ*) of the ZnO films with different thicknesses on Ru(0001). (B) H adsorption energy on ZnO/Ru(0001) *versus* the thickness of ZnO films. As a comparison, those on ZnO/Au(111) films are also given in open triangles.

Following the procedure of the ZnO/Au(111) interface, we identified the most favourable ZnO/Ru(0001) surface structure (Fig. S5†). Similar to free-standing ZnO and ZnO/Au(111), G-ZnO/Ru(0001) is more stable than W-ZnO/Ru(0001) for thin films, while the latter becomes more preferable for thick films (Fig. 5A). The critical thickness is between 5 and 6 ML, which is 3 and 2 ML thinner than that of free-standing ZnO and ZnO/Au(111), respectively. Intriguingly, W-ZnO/Ru(0001) structures prefer to expose the Zn-terminated W-ZnO(0001) surface, for which Δ*μ* is 0.04 eV/ZnO lower than that of O-terminated W-ZnO(0001̄). Such a preference of surface terminations is opposite to that of ZnO/Au(111), probably because of the stronger interaction of Ru with the interface O atoms than that of Au. We expect that this difference may substantially impact the surface reactivity and its tuning.

Indeed, as shown in Fig. 5B, the hydrogen adsorption energy on W-ZnO/Ru(0001) with Zn termination is weakened by 1.3 eV in comparison with that on W-ZnO/Au(111) with O termination, *i.e.* −0.4 *versus* −1.7 eV. Thus, surface reactivity can be substantially tuned by surface termination that is controlled by varying the chemical nature of the support. It is worth noting that the notable weakening of hydrogen adsorption on W-ZnO/Ru(0001), in comparison with that on free-standing W-ZnO and W-ZnO/Au(111), does not lead to an increased hydrogen dissociation barrier, but rather a decreased barrier (from 1.04 and 0.86 eV to 0.63 eV in Fig. S6A and B†). This is because Zn sites promote H_2_ activation, in comparison to O sites. The important implication of such an opposite trend between surface reactivity and reaction kinetics is manifested by the broken BEP relationship, or more precisely the establishment of a new BEP relationship. This is further confirmed on G-ZnO/Ru(0001), for which hydrogen adsorption is in the range of −0.26 to −0.77 eV. While such a surface reactivity is very similar to that of W-ZnO/Ru(0001), there is a ∼1.5 eV difference in the H_2_ dissociation barrier (Fig. 1 and S6C†). Thus, varying the ZnO film thickness and the chemical nature of the support not only leads to flexible tuning of surface reactivity and the associated kinetic barrier, but also changes the scaling relationship between surface reactivity and reaction kinetics, *i.e.* so-called breaking the BEP relationship.

It is worth noting that metal substrates substantially interact with ZnO through interfacial bonding (Fig. S7A†) where charge transfer occurs. As Ru shows a stronger affinity (−3.16 eV evaluated by the standard formation enthalpy^37^) to O than Au (−0.02 eV), it results in differences in the amount and the direction of charge transfer, *i.e.* 0.01|*e*|/ZnO from ZnO to Au(111) *vs.* 0.05|*e*|/ZnO from Ru(0001) to ZnO in the case of the 1 ML film.^22^ These differences lead to more rumpled G-ZnO/Ru and Zn-termination of W-ZnO/Ru, in comparison with ZnO/Au (Fig. S7B and C†), which are the fundamental reasons for the support dependence.

### Perspectives of 2D oxide design towards H_2_ activation

H adsorption energy serves as a key descriptor for H-involved reactions in heterogeneous catalysis, such as CO/CO_2_ hydrogenation,^20,38^ alkyne semi-hydrogenation,^39,40^ methane activation *via* a radical mechanism,^8,41^ among others. For these reactions, different but specific H adsorption is required to optimize catalytic activity. The flexible large-scale reactivity tuning of 2D ZnO makes it a candidate for multi-functional catalysts for a variety of reactions. Also, for a multiple-step reaction, improving the energetics of one elementary step may worsen the energetics of another elementary step on the same catalyst. On the other hand, the flexible large-scale reactivity tuning of 2D ZnO makes it possible to enhance the overall reaction by simultaneously optimizing multiple elementary steps.

## Conclusions

By employing DFT calculations, we demonstrate the possibility of flexible large-scale tuning of surface reactions, which is usually achievable only through multiple compounds, by manipulating the phase, thickness and support of 2D ZnO films. Using the H adsorption energy as a descriptor, we show that, for free-standing ZnO films, up to 3 eV tuning of surface reactivity can be achieved by controlling the phase of ZnO films. Such large-scale tuning is because the polar wurtzite phase possesses a higher surface reactivity than the nonpolar graphene phase with coplanar Zn and O atoms, which is intrinsically due to lowering of the coordination of surface O. We further demonstrate that the reactivity of film phases can be tuned by forming inverse ZnO/metal interfaces and by varying the ZnO thickness. For ZnO/Au(111), the presence of the substrate leads to 1–2 eV stability of hydrogen adsorption on the graphene phase in comparison with the free-standing ZnO, accompanied by a corresponding reduction in the kinetic barrier of H_2_ dissociation. Such a stabilization not only results in more moderate binding of hydrogen on the surface, but also finer reactivity tuning *i.e.* a few tenths of eV by varying the film thickness. For ZnO/Ru(0001) with preferential Zn termination for the wurtzite phase, while the hydrogen adsorption is weakened by around 1.3 eV in comparison with ZnO/Au(111) with preferential O termination, the kinetic barrier of H_2_ dissociation is reduced by over 0.2 eV. Such a trend suggests that varying the chemical nature of the support not only leads to the tuning of surface reactivity, but also a change of the scaling relationship between surface reactivity and reaction kinetics, *i.e.* breaking the BEP relationship. Thus, the current work provides an alternative strategy for flexible large-scale tuning of surface reactivity and reaction kinetics, which is of significance for the development of multi-functional catalysts.

## Methods

### Computational parameters

DFT calculations were implemented using a plane wave basis set in the Vienna *Ab initio* Simulation Package (VASP 5.4).^42,43^ The exchange-correlation energy was evaluated using the Perdew–Burke–Ernzerhof (PBE)^44^ functional within the generalized gradient approximation (GGA).^45^ The projected-augmented wave (PAW)^46^ pseudopotentials were utilized to describe the core electrons, and a cutoff energy of 400 eV was used for the plane-wave expansion. The following valence electron configurations were included in the self-consistent field calculations: Zn (3d^10^ and 4s^2^), O (2s^2^ and 2p^4^), Au (5d^10^ and 6s^1^), Ru (4d^7^ and 5s^1^), and H (1s^1^). In addition, the van der Waals (vdW) dispersion forces were corrected by adopting vdW-DF (optPBE) functionals,^47^ which showed excellent performance toward a highly accurate description of oxides.^48^ The energies and residual forces were converged to 10^−5^ eV and 0.02 eV Å^−1^, respectively. The transition states were searched using the climbing-image nudged elastic band (CI-NEB) technique with only one imaginary frequency determined.

### Models

The lattice constants of wurtzite ZnO were calculated to be *a* and *b* = 3.288 Å and *c* = 5.347 Å, agreeing well with the experimental results.^49^ The lattice constant of Au is *a* = 4.192 Å and those of Ru are *a* = 2.772 Å and *c* = 4.304 Å. For free-standing (FS) ZnO, we used a (2 × 2) supercell along the wurtzite ZnO[0001] orientation and a (2 × 1) supercell along the body-centered-tetragonal (BCT) ZnO[100] orientation to study the adsorption. The 4 × 4 × 1 *k*-point grids were used for Brillouin zone sampling. For Au(111)-supported ZnO, the used supercells were as follows: (√28 × √28)/(√39 × √39), (√3 × √3)/(2 × 2), (√7 × √7)/(3 × 3), (√13 × √13)/(4 × 4), (4 × 4)/(√19 × √19), and (√27 × √27)/(√31 × √31) with interface Zn/Au ratios of 0.72, 0.75, 0.78, 0.81, 0.84, and 0.87, respectively. The corresponding *k*-point grids were 3 × 3 × 1, 12 × 12 × 1, 9 × 9 × 1, 6 × 6 × 1, 6 × 6 × 1, and 4 × 4 × 1 in sequence. Likewise, the supercells for ZnO/Ru(0001) were constructed including (4 × 4)/(√27 × √27), (4 × 4)/(5 × 5), (3 × 3)/(√13 × √13), (√28 × √28)/(√39 × √39), and (√3 × √3)/(2 × 2) with interface Zn/Ru ratios of 0.59, 0.64, 0.69, 0.72, and 0.75, respectively. The corresponding *k*-point grids within the Monkhorst–Pack scheme were 5 × 5 × 1, 4 × 4 × 1, 6 × 6 × 1, 6 × 6 × 1, and 12 × 12 × 1 in sequence. Here, the substrates were treated as rigid with their bulk lattices. Three atomic layer metal slabs were utilized to mimic the substrate and the bottom two were constrained. The slabs were separated by a more than 12 Å vacuum layer.

## Data availability

Further datasets supporting this article have been uploaded as part of the ESI.†

## Author contributions

L. L. conducted the theoretical calculations. Z. Z. analyzed the computational results and the suggestions of writing. Z. Z and L. L. wrote and edited the manuscript with input from all authors. Q. F. and X. B. designed and supervised the project.

## Conflicts of interest

The authors declare no conflict of interest.

## Supplementary Material

SC-012-D1SC04428A-s001
