# Supplementary material for: Achieving flexible large-scale reactivity tuning by controlling the phase, thickness and support of two-dimensional ZnO
Source: Chem Sci. 2021 Nov 4;12(46):15284–90. doi: 10.1039/d1sc04428a (PMC8635171; doi:10.1039/d1sc04428a)
Supplement: SC-012-D1SC04428A-s001 [file SC-012-D1SC04428A-s001.pdf]

Electronic Supplementary Material (ESI) for Chemical Science.
This journal is © The Royal Society of Chemistry 2021

Supporting Information:

Achieve flexible large-scale reactivity tuning by controlling phase, thickness and support of two-dimensional ZnO

Le Lin,[abcd] Zhenhua Zeng,[*e] Qiang Fu[*b] and Xinhe Bao[b]

[a] Shanghai Advanced Research Institute, Chinese Academy of Sciences, Shanghai 201203, China
[b] State Key Laboratory of Catalysis, Dalian Institute of Chemical Physics, Chinese Academy of Sciences, Dalian 116023, China
[c] School of Physical Science and Technology, ShanghaiTech University, Shanghai 201210, China
[d] University of Chinese Academy of Sciences, Beijing 100049, China
[e] Davidson School of Chemical Engineering, Purdue University, West Lafayette, IN 47907, USA

The electronic Supporting Information (ESI) includes the involved formulae, figures, tables, and notes, as below.

1. Formulae

(1) The relative chemical potential ($\Delta\mu$):

$$\Delta\mu = \frac{1}{N}\left(E_{FS-ZnO} - NE_{bulk-ZnO}\right)$$

$$\Delta\mu = \frac{1}{N}\left(E_{ZnO/M} - E_{M} - NE_{bulk-ZnO}\right)$$

where $E_{\text{FS-ZnO}}$, $E_{\text{ZnO/M}}$, E_{M}, and $E_{\text{bulk-ZnO}}$ are the total energies of the free-standing ZnO film, supported ZnO/M, clean substrate (M), and bulk ZnO counterpart, respectively. N is the number of the ZnO pairs.

(2) The adsorption energy of atomic hydrogen (E_{ads}):

$$E_{ads} = \left(E_{nH*} - E_{*} - \frac{n}{2}E_{H_2}\right)/n$$

where E_{nH*}, E_{*}, and E_{H_2} are the energies of the n*H adsorbed surface, clean surface, and gaseous H_2 molecule.

(3) Zero point energy (E_{ZPE}) correction for gaseous H_2 molecule and adsorbed H*:

$$E_{H_2} = E_{elec}(H_2) + E_{ZPE}$$

$$E_{nH*} = E_{elec}(nH*) + E_{ZPE}$$

where E_{elec} is the energy obtained directly from DFT calculation.

(4) The intrinsic strain (τ) of ZnO films relative to the wurtzite bulk is calculated by

$$\tau = \frac{a_{ZnO-film} - a_{bulk-ZnO}}{a_{bulk-ZnO}} \times 100\%$$

where $a_{\text{ZnO-film}}$ and $a_{\text{bulk-ZnO}}$ are the lattice constants of the ZnO film and the wurtzite ZnO, respectively.

2. Note

When formation of the ZnO/Au(111) interface, concomitant corrugation of the ZnO layer is usually inevitable owing to lattice mismatch. The sites presented on the surface are uneven and need to be screened for ascertaining the potential active sites. As shown in **Fig. S8†**, we choose three most active O sites to calculate the average energies of H adsorption on the surface of ZnO/Au(111) models, corresponding to an H coverage of 0.23 (ϑ). Accordingly, two most active O sites are used for ZnO/Ru(0001) models (ϑ = 0.22).

3. Figures and Tables

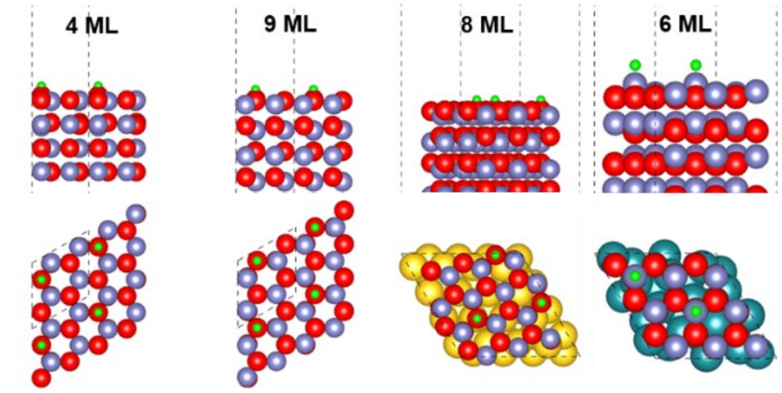

Fig. S1 Configurations of atomic hydrogen adsorbed on the surface O/Zn sites for 4 ML G-ZnO, 9 ML W-ZnO(000$\bar{1}$), 8 ML W-ZnO(000$\bar{1}$)/Au(111), and 6 ML W-ZnO(0001)/Ru(0001). H: green; O: red; Zn: light blue; Au: golden; Ru: olive.

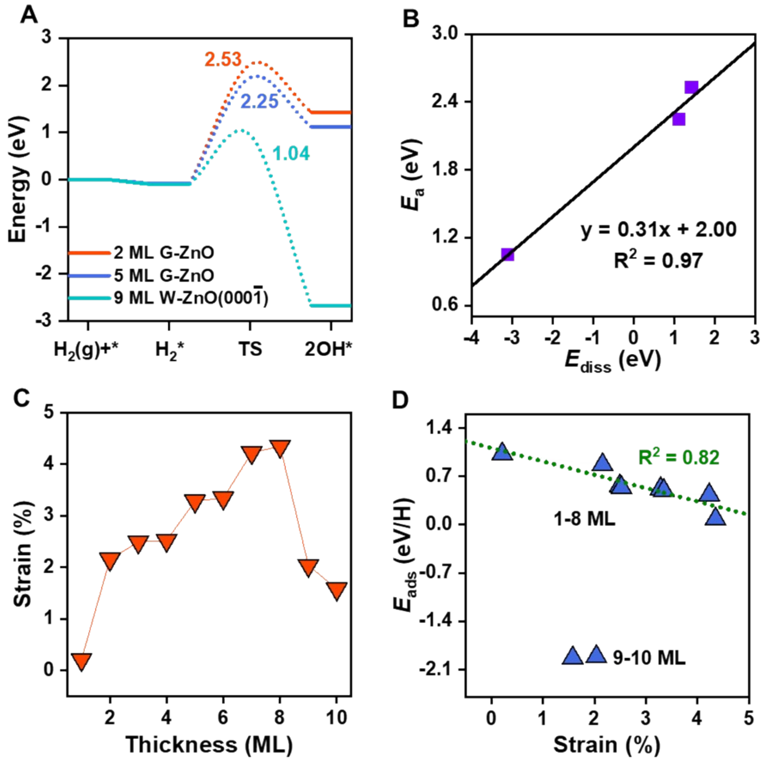

Fig. S2 H_2 dissociation on the surfaces of free-standing ZnO films, including the 2 ML and 5 ML G-ZnO, and 9 ML W-ZnO(000$\bar{1}$). (**A**) Potential energy diagrams of H_2 dissociation on the surfaces. The inset value denotes the energy barrier calculated by the energy of the TS (transition state) subtracting that of the IS (initial state, i.e. the H_2* state). (**B**) The BEP relationship between the barrier (E_a) and the dissociative adsorption energy (E_{diss}, referenced to the gaseous H_2). (**C**) Intrinsic strain (vs. the wurtzite bulk) on ZnO films as a function

of the thickness. (**D**) H adsorption energies (E_{ads}) as a function of intrinsic strain on ZnO films, where the fitting line for 1–8 ML denotes a plausible scaling relationship between E_{ads} and strain.

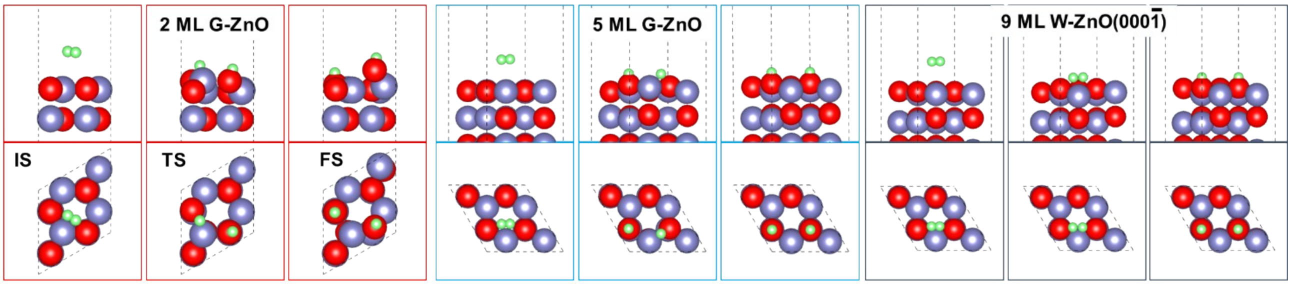

Fig. S3 Top (lower) and side (upper) views of H_2 dissociation on the surfaces of the 2 ML and 5 ML G-ZnO, and 9 ML W-ZnO(000$\bar{1}$). The IS, TS, and FS (final state, i.e. the 2H* state) are included, corresponding to Fig. S2. H: green; O: red; Zn: light blue.

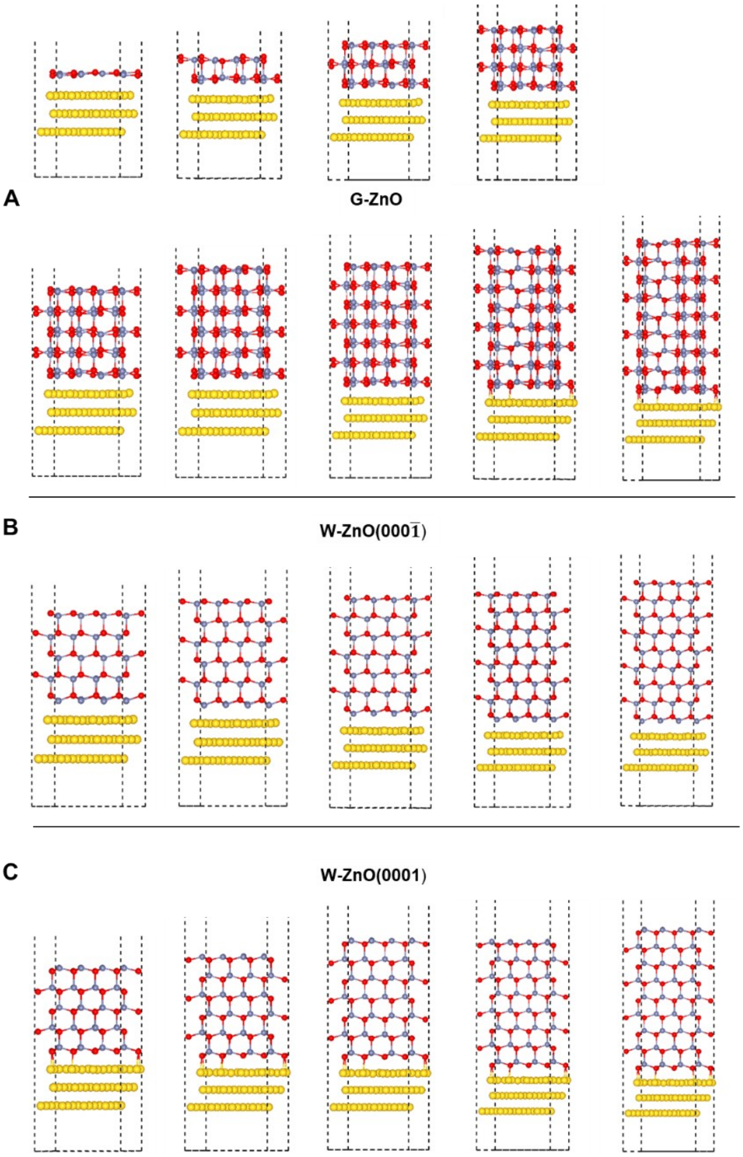

Fig. S4 Film stability and surface termination for the ZnO-($\sqrt{13} \times \sqrt{13}$)/Au(111)-(4 × 4) moiré pattern. (**A**) G-ZnO phase (1–9 ML), (**B**) O-terminated W-ZnO(000$\bar{1}$) (5–9 ML), and (**C**) Zn-terminated W-ZnO(0001) (5–9 ML). O: red; Zn: light blue; Au: golden.

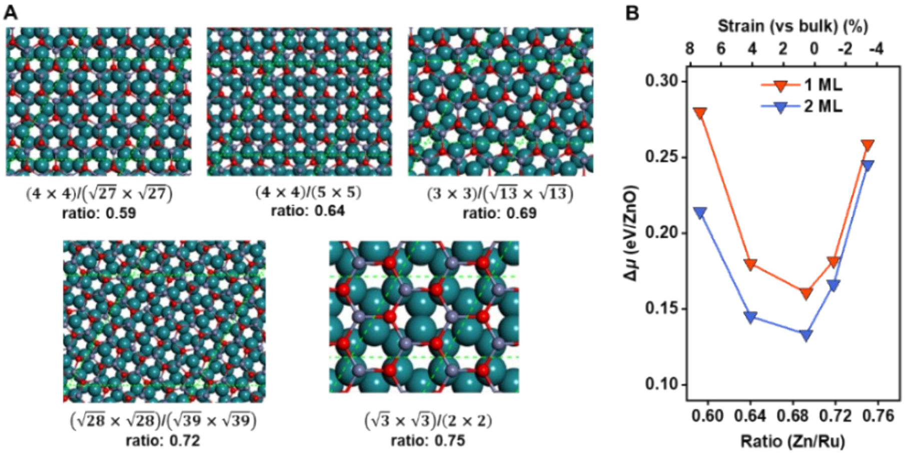

Fig. S5 Interface models and the energetics of G-ZnO films on Ru(0001). (**A**) Various moiré patterns of monolayer ZnO films supported on Ru(0001). The green dashed lines denote the boundaries of the unit cells. O: red; Zn: light blue; Ru: olive. (**B**) The relative chemical potential ($\Delta\mu$), with respect to the bulk ZnO and Ru substrate, as a function of the interface Zn/Ru ratio (the lower x-axis) and the intrinsic strain on the ZnO overlayer (the upper x-axis), for 1–2 ML.

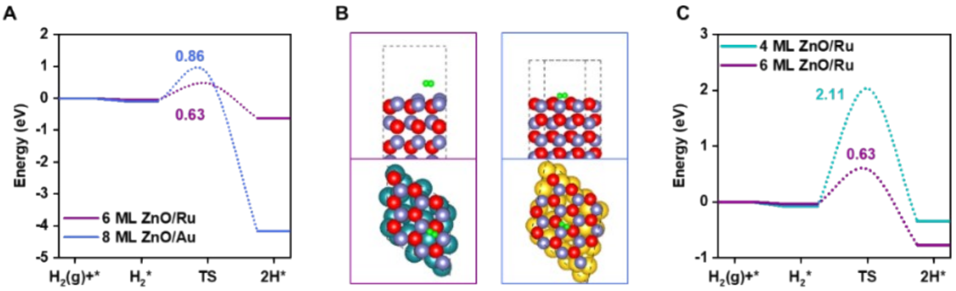

Fig. S6 H_2 dissociation on the surfaces for Au(111) and Ru(0001) supported ZnO films. (**A**) H_2 dissociation on the surface O sites for 8 ML W-ZnO(000$\bar{1}$)/Au(111) and the surface Zn sites for 6 ML ZnO(0001)/Ru(0001), respectively. The inset values represent the energy barriers. (**B**) Configurations of the corresponding TS structures. H: green; O: red; Zn: light blue; Au: golden; Ru: olive. (**C**) H_2 dissociation on the surface of 4 ML G-ZnO/Ru(0001) where 6 ML W-ZnO/Ru(0001) is given as a comparison.

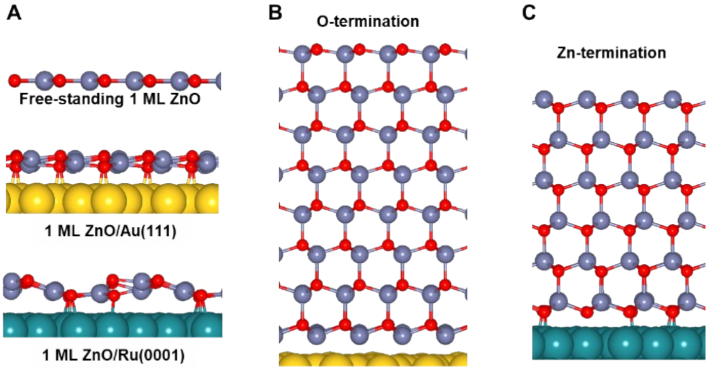

Fig. S7 Scheme for the monolayer ZnO, the 8 ML W-ZnO(000$\bar{1}$)/Au(111), and the 6 ML W-ZnO(0001)/Ru(0001) films. O: red; Zn: light blue; Au: golden; Ru: olive.

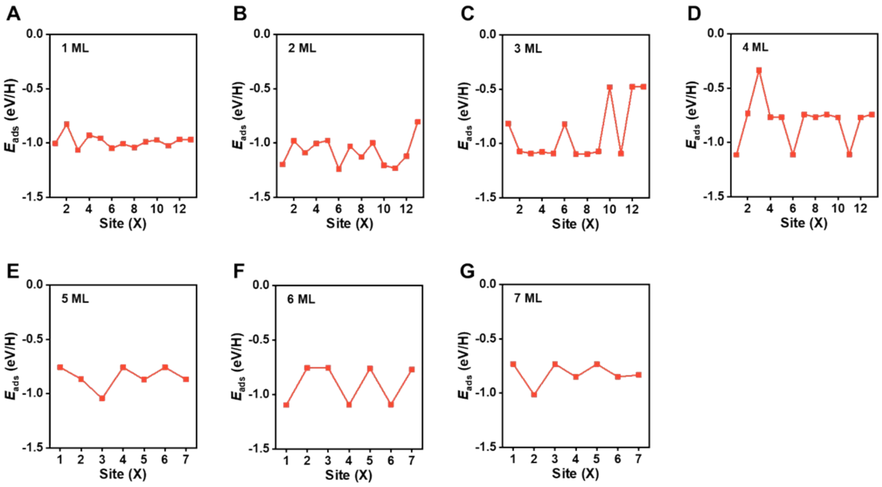

Fig. S8 Adsorption energies of atomic hydrogen on surface O sites of G-ZnO/Au(111) films from 1 to 7 ML (A-G). Note: The coverage (ϑ) is 0.08. This shows the unevenness of the activity of the surface O sites. Nevertheless, we choose the three most active O sites to obtain the average H adsorption in the main text, corresponding to a coverage of 0.23 (an example of H adsorbed on 8 ML W-ZnO/Au is shown in **Fig. S1**), so as to have a fair comparison with free-standing ZnO films (ϑ = 0.25).

Tab. S1 Moiré patterns of ZnO films supported on Au(111) with interface Zn/Au ratios from 0.72 to 0.87. The angle (α) between the film and the substrate are given.

ZnO film	Substrate	Ratio	α (°)	Strain (%)
√28 × √28	√39 × √39	0.72	3.0	6.4
√3 × √3	2 × 2	0.75	30.0	4.1
√7 × √7	3 × 3	0.78	19.1	2.2
√13 × √13	4 × 4	0.81	13.9	0.0
4 × 4	√19 × √19	0.84	23.4	-1.8
√27 × √27	√31 × √31	0.87	21.0	-3.4

Tab. S2 Moiré patterns considered for ZnO films supported on Ru(0001) with interface Zn/Ru ratios from 0.59 to 0.75. The angle (α) between the film and the substrate are given.

ZnO film	Substrate	Ratio	α (°)	Strain (%)
4 × 4	√27 × √27	0.59	30.0	7.5
4 × 4	5 × 5	0.64	0	3.5
3 × 3	√13 × √13	0.69	13.9	-0.5
√28 × √28	√39 × √39	0.72	3.0	-2.3
√3 × √3	2 × 2	0.75	30.0	-4.4

Tab. S3 Zero vibrational energy (E_{ZPE}) corrections of the gas and the adsorbates. (unit: eV)

Species	E_{ZPE}
H_2(g)	0.27
H*(O)	0.31
H*(Zn)	0.16
